# The α6 GABA_A_ Receptor Positive Allosteric Modulator DK-I-56-1 Reduces Tic-Related Behaviors in Mouse Models of Tourette Syndrome

**DOI:** 10.3390/biom11020175

**Published:** 2021-01-28

**Authors:** Roberto Cadeddu, Daniel E. Knutson, Laura J. Mosher, Stefanos Loizou, Karen Odeh, Janet L. Fisher, James M. Cook, Marco Bortolato

**Affiliations:** 1 Department of Pharmacology and Toxicology, College of Pharmacy, University of Utah, Salt Lake City, UT 84112, USA; roberto.cadeddu@utah.edu (R.C.); laura.mosher@utah.edu (L.J.M.); stefanos.loizou@utah.edu (S.L.); karen.odeh@utah.edu (K.O.); 2 Department of Chemistry and Biochemistry, Milwaukee Institute for Drug Discovery, University of Wisconsin-Milwaukee, Milwaukee, WI 53211, USA; knutsond@uwm.edu (D.E.K.); capncook@uwm.edu (J.M.C.); 3 Department of Pharmacology, Physiology and Neuroscience, School of Medicine, University of South Carolina, Columbia, SC 29209, USA; Janet.Fisher@uscmed.sc.edu

**Keywords:** DK-I-56-1, Tourette syndrome, tics, spontaneous eyeblinks, GABA_A_ receptor

## Abstract

Tourette syndrome (TS) is a disabling neurodevelopmental disorder characterized by multiple, recurrent tics. The pharmacological treatment of TS is currently based on dopaminergic antagonists; however, these drugs are associated with extrapyramidal symptoms and other serious adverse events. Recent evidence suggests that positive allosteric modulators (PAMs) of GABA_A_ receptors containing α6 subunits (α6 GABA_A_Rs) oppose the behavioral effects of dopamine. Building on this evidence, in the present study, we tested the efficacy of DK-I-56-1, a highly selective PAM for α6 GABA_A_Rs, in mouse models of TS exhibiting tic-related responses. DK-I-56-1 significantly reduced tic-like jerks and prepulse inhibition (PPI) deficits in D1CT-7 transgenic mice, a well-documented mouse model of TS. DK-I-56-1 also prevented the exacerbation of spontaneous eyeblink reflex induced by the potent dopamine D_1_ receptor agonist SKF 82958, a proxy for tic-like responses. We also showed that both systemic and prefrontal cortical administration of DK-I-56-1 countered the PPI disruption caused by SKF 82958. Although the effects of DK-I-56-1 were akin to those elicited by dopaminergic antagonists, this drug did not elicit extrapyramidal effects, as measured by catalepsy. These results point to α6 GABA_A_R PAMs as promising TS therapies with a better safety profile than dopaminergic antagonists.

## 1. Introduction

Tics are semi-voluntary movements or utterances, typically executed in a contextually inappropriate and repetitive fashion. Although tics vary greatly in duration and complexity, they are often manifested as clonic, sudden spasms, such as eyeblinks, facial grimaces, head jerking, throat clearing, and shoulder shrugging. The most disabling tic disorder, Tourette syndrome (TS), is a neurodevelopmental illness characterized by multiple motor and at least one phonic tic for longer than one year [1]. TS is also characterized by other phenotypic alterations, such as information processing deficits [2]; in particular, TS patients exhibit alterations of prepulse inhibition (PPI) of the startle reflex [3], an operational index of sensorimotor gating. 

Although the causes of TS remain partially elusive, ample evidence has shown that this disorder has a strong genetic predisposition [4]. Its etiology is also contributed by multiple environmental factors, including prenatal and perinatal adverse events [5]. The interaction of these factors is interpreted to lead to dysregulated connectivity of the cortico-striato-thalamo-cortical connectivity [6,7,8,9] and contributed by alterations of the dopaminergic system [10,11]. Accordingly, the mainstay of the pharmacological therapy of TS and other chronic tic disorders is based on dopamine D_2_ receptor antagonists, such as haloperidol (HAL) and pimozide [12]; in addition, recent trials have shown that the highly selective D_1_ receptor antagonist ecopipam produces similar therapeutic effects [13]. However, dopamine receptor blockers are not optimal therapies due to their significant adverse events, such as extrapyramidal symptoms [14], as well as their inconsistent efficacy and limited therapeutic compliance.

**Figure 1:**
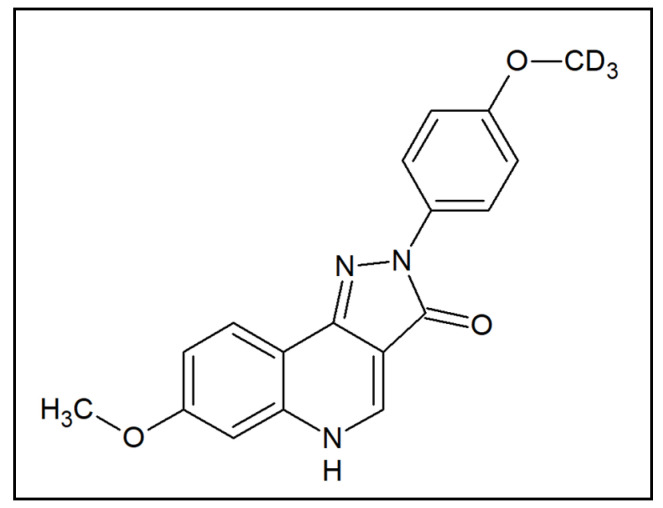
Chemical structure of DK-I-56-1.

Emerging preclinical evidence suggests that the activation of GABA_A_ receptors containing α6 subunits (α6 GABA_A_Rs) may be a potential therapeutic agent for tic disorders. Specifically, it was demonstrated that positive allosteric modulators (PAMs) of these receptors oppose the PPI deficits induced by the potent dopamine releaser methamphetamine [15]. Although these data are promising, PPI deficits are not pathognomonic of TS, but are exhibited across a wide range of heterogeneous neuropsychiatric disorders, including schizophrenia, mania, and obsessive-compulsive disorder (OCD) [16]. To understand whether this category of compounds may also reduce tics, the present study was designed to assess the therapeutic potential of the pyrazoloquinolinone DK-I-56-1 (7-methoxy-2-(4-methoxy-*d*_3_-phenyl)-2,5-dihydro-3*H*-pyrazolo[4,3-*c*]quinolin-3-one) (Figure 1), a highly selective and potent PAM of α6 GABA_A_Rs [17]. This compound was developed as a stable analog of the α6 GABA_A_R PAM PZ-II-029, with enhanced bioavailability and a similar lack of cytotoxic, hepatotoxic, or sedative effects [17,18].

Rodent models of TS are highly valuable tools for the identification of novel therapeutic targets [19]. Interpreting which motoric phenomena in animal models can be reliably likened to tics is a significant challenge due to the heterogeneous nature of these manifestations in patients and the incomplete knowledge of their neurobiological mechanisms [20]. Thus, predictions on the therapeutic potential of investigational drugs in TS should be preferably based on multiple, complementary models, capturing different aspects of this disorder. In view of this premise, we tested the effects of DK-I-56-1 in two complementary models, D1CT-7 mice and the exacerbation of spontaneous eyeblink by D_1_ receptor activation, which have high face validity with respect to human tics, insofar as they exhibit sudden spontaneous movements highly isomorphic with these manifestations. 

D1CT-7 mice are a transgenic line generated through the attachment of a neuropotentiating cholera toxin to the D_1_ dopamine receptor promoter; these animals display tic-like manifestations, consisting of brief, sudden axial jerks [21,22]. We previously reported that exposing these animals to brief stressors dramatically increases these manifestations and elicits PPI deficits [23]. These characteristics confer high face and predictive validity for TS to D1CT-7 mice. 

Spontaneous eyeblinks are regarded as a reliable index of dopaminergic activity in the brain [24] and are significantly exacerbated by the activation of D_1_ receptors [25]. Several premises point to these manifestations as the closest phenomenological proxies of tics: first, they represent the most common type of tics; second, both spontaneous eyeblinks and tics are preceded by similar premonitory urges, based on common neurobiological substrates [26]; third, TS patients display a significant increase in spontaneous eyeblink frequency [27,28] and a lower ability to suppress eyeblinks [29]. Notably, D_1_ receptor activation in mice is also conducive to other TS-related responses, including grooming stereotypies and PPI deficits [30,31].

To capture both the therapeutic potential and the possible extrapyramidal effects of DK-I-56-1, we also tested its effects on the PPI deficits displayed by both mouse models and compared its effects with those elicited by D_1_ and D_2_ receptor antagonists, also with respect to catalepsy, a well-established paradigm to measure extrapyramidal symptoms.

## 2. Materials and Methods

Animals. Adult C57BL/6J mice (3–4 months old, 25–35 g) were purchased from the Jackson Laboratory (Bar Harbor, ME, USA) and acclimated to housing facilities for 7–10 days. D1CT-7 mice and their wild-type (WT) littermates were bred in the husbandry facilities of the University of Utah and genotyped as previously described [23]. Mice were group-housed (3/cage), with food and water ad libitum. Housing facilities were maintained at 22 °C with a 12-h light/dark cycle (lights on from 6:00 AM to 6:00 PM). Experimental manipulations were carried out between 9:00 AM and 4:00 PM. Handling and experimental procedures were performed in compliance with the National Institute of Health guidelines and approved by the Institutional Animal Care and Use Committee at the University of Utah.

Drugs. For systemic injections, DK-I-56-1 was synthesized as previously described [17] and dissolved in 2.5% DMSO, 2.5% Tween 80, and 0.9% NaCl. For local intracerebral infusions, DK-I-56-1 was dissolved in DMSO. SKF 82958, SCH 23390, and HAL were purchased from Sigma-Aldrich (St. Louis, MO, USA) and dissolved as previously described [32].

Experimental procedures. In the first series of experiments, we tested the effects of DK-I-56-1 on tic-like behaviors and PPI deficits in D1CT-7 and WT mice exposed to spatial confinement (as this stressor has been shown to dramatically augment tic-like responses and disrupt sensorimotor gating). Following a 30-min session of spatial confinement (during which tic-like responses and other spontaneous behaviors were monitored), mice underwent startle and PPI testing, as previously described [23,33]. 

In the second series of experiments, we studied how DK-I-56-1 affected the enhancement of spontaneous eyeblinks, grooming stereotypies, and PPI deficits caused by the D_1_ dopamine receptor agonist SKF 82958 in C57BL/6 mice. Finally, to chart the neuroanatomical substrates responsible for the effects of DK-I-56-1, we tested the effects of local injections of DK-I-56-1 in the medial prefrontal cortex and nucleus accumbens, the two key brain areas involved in PPI deficits induced by dopamine receptor agonists. 

Throughout the study, the effects of DK-I-56-1 were compared with those of the prototypical D_1_ and D_2_ receptor antagonists SCH23390 and haloperidol. All experiments were performed in counterbalanced order to minimize potential differences in testing time. 

Spatial confinement and evaluation of tic-like responses and spontaneous behaviors in D1CT-7 mice. Spatial confinement within the home-cage environment was used as an environmental stressor to elicit tic-like responses and PPI deficits in D1CT-7 mice, as previously reported [33]. Briefly, mice were single-housed for five days before testing to allow for the establishment of territorial behavior and to maximize the stressfulness of confinement. Immediately after treatment, mice were confined within a clear, bottomless Plexiglass cylinder (diameter: 10 cm; height: 30 cm), inside their home cage. Behaviors were video-recorded for the entire session. Only the last 20-min sessions were scored (to allow neophobia to subside and drugs to be distributed in the brain). Tic-like behaviors (rapid motor outburst, consisting of twitches of the head, and/or muscle contractions of the body), digging, grooming, and rearing behaviors were scored by trained observers blind to genotype and treatments, using Behavioral Tracker 1.5 (http://behaviortracker.com) software. Treatments were randomly assigned to each experimental group.

**Figure 2:**
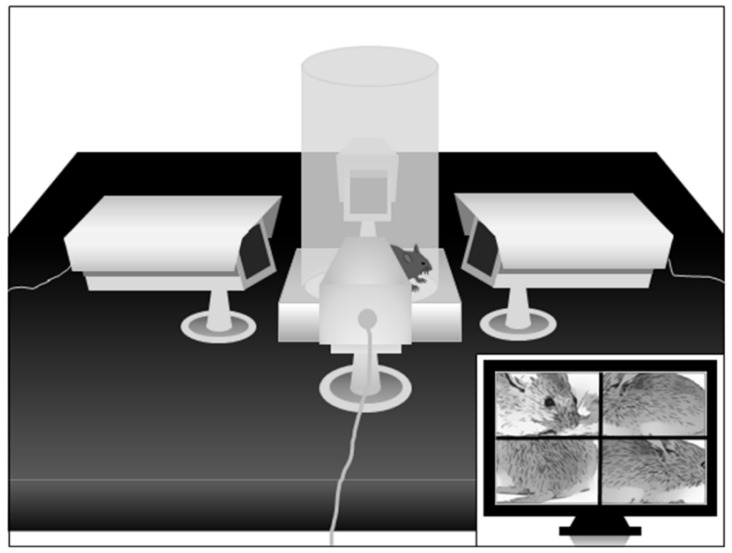
Schematic representation of the apparatus to study spontaneous eyeblinks in mice. For further details, see text.

Spontaneous eye blinking. Eye blinking was studied by placing mice in the same cylinder used for spatial confinement, mounted on a square platform adjacent to four video cameras placed on each of the four sides in close proximity to the cylinder, as shown in Figure 2. This configuration allows experimenters to monitor eyeblinks remotely and continuously, in a non-invasive fashion, and without the employment of head restraint bars. To minimize stress, animals were exposed to the apparatus for 3 days before testing, and the actual testing session was limited to 5 min. Eyeblinks and grooming stereotypies were scored by trained observers blind to treatment groups, using Behavioral Tracker. Treatments were randomly assigned to each experimental group.

PPI. Startle testing was conducted as previously described [34]. Briefly, the apparatus used to measure startle reflexes (SR-LAB; San Diego Instruments, San Diego, CA, USA) consisted of six Plexiglas cages (diameter: 5 cm) in sound-attenuated chambers with fan ventilation. Each cage was mounted on a piezoelectric accelerometric platform connected to an analog-digital converter. The response to each stimulus was recorded as 65 consecutive 1-ms readings. A dynamic calibration system was used to ensure comparable sensitivities across chambers. The startle testing protocol featured a 70-dB background white noise and consisted of a 5-min acclimatization period, followed by three consecutive blocks of pulse, prepulse + pulse, and “no stimulus” trials. During the first and the third block, mice received only five pulse-alone trials of 115 dB. Conversely, in the second block, mice were exposed to a pseudorandom sequence of 50 trials, consisting of 12 pulse-alone trials, 30 trials of pulse preceded by 73, 76, or 82-dB prepulse intensities (10 for each level of prepulse loudness), and eight no stimulus trials, where only the background noise was delivered. Intertrial intervals were selected randomly between 10 and 15 s. Sound levels were assessed using an A-scale setting. Percent PPI (%PPI) was calculated using the formula: [1 − (mean startle in “prepulse-pulse” trials/mean startle in “pulse-alone” trials)] × 100. The first five pulse-alone bursts were excluded from the calculation. As no interaction between prepulse levels and treatment was found in the statistical analysis, %PPI values were collapsed across prepulse intensities to represent average %PPI.

Stereotaxic cannulation. Stereotaxic surgery was performed with a protocol based on our previous publication [35], adapted for mice. Briefly, mice were anesthetized with ketamine/xylazine (80 mg/kg; 20 mg/kg, IP) and placed in a stereotaxic apparatus (Kopf, Tujunga, CA, USA). Bilateral craniotomies were performed above the target sites, and stainless steel cannulae were lowered into place and implanted using dental cement. The lengths of the cannulae were selected to end 0.5 mm above the targeted areas, with the corresponding injector projecting 1 mm beyond the guide tip. The target locations for cannulation from bregma were the medial prefrontal cortex (AP +1.8 mm, ML ±0.3 mm, DV −2.5 mm) and nucleus accumbens (AP +1.4 mm, ML ±0.5 mm, DV −4.3 mm). These locations were selected based on their well-characterized role in regulating sensorimotor gating by dopaminergic agonists. Coordinates were taken from bregma, according to the stereotaxic brain atlas by Franklin and Paxinos [36]. Mice were given antibiotic therapy for two days (enrofloxacin, Bayer HealthCare, Shawnee Mission, KS, USA) and allowed to recover in their home cages for seven days before testing.

Bar test. Catalepsy was measured by means of the bar test, performed as described previously [37]. Briefly, the forepaws of the mouse were placed on a bar fixed at the height of 5 cm above the working surface. The latency to descend from the bar with both paws was calculated.

Statistical analysis. Normality and homoscedasticity of data distribution were verified using Kolmogorov–Smirnov and Bartlett’s tests. Statistical analyses of parametric data were performed by one- or two-way ANOVA, as appropriate, followed by Newman–Keuls test for post-hoc comparisons. Statistical significance was set at *p* < 0.05. 

## 3. Results

**Figure 3:**
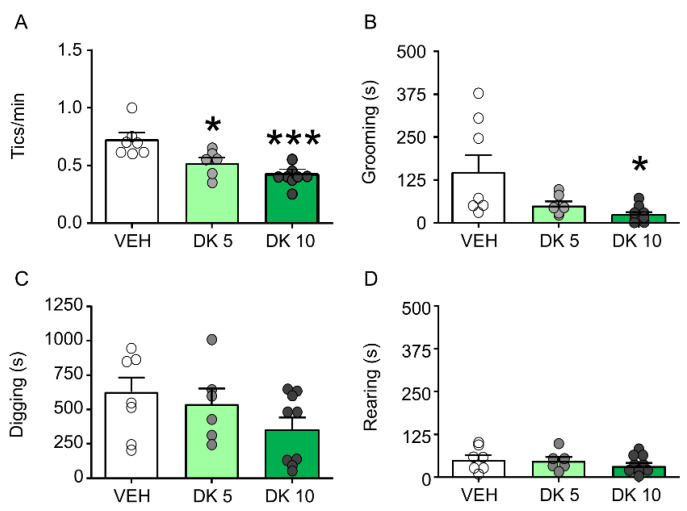
Effects of DK-I-56-1 (DK) on Tourette Syndrome-related motoric responses in D1CT-7 mice exposed to spatial confinement. DK significantly reduced (**A**) tic-like responses (F(2,15) = 9.66, *p* = 0.002) and the duration of (**B**) grooming behavior (F(2,19) = 4.48; *p* = 0.03), but not (**C**) digging or (**D**) rearing. All analyses were performed by one-way ANOVAs. Significance refers to the results of post-hoc comparisons. * *p* < 0.05; *** *p* < 0.001 in comparison with vehicle (VEH). *n* = 6–8/group. Doses are indicated in mg/kg (IP). For further details, see text.

DK-I-56-1 dose-dependently reduces tic-like behaviors and PPI deficits in D1CT-7 mice. We first tested the effects of DK-I-56-1 (5, 10 mg/kg, IP) on tic-like responses in D1CT-7 mice subjected to spatial confinement. As shown in Figure 3, DK-I-56-1 was significantly effective in reducing tic-like responses (Figure 3A) and grooming behavior (Figure 3B), but not digging (Figure 3C) or rearing (Figure 3D). 

**Figure 4:**
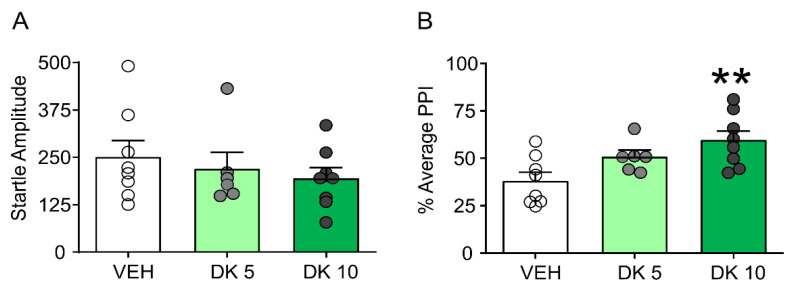
Effects of DK-I-56-1 (DK, 5, 10 mg/kg, IP) on (**A**) startle response and B) prepulse inhibition (PPI) (**B**) in D1CT-7 mice exposed to spatial confinement. DK had no effects on startle amplitude but restored PPI (F(2,19) = 6.05; *p* = 0.009). All analyses were performed by one-way ANOVAs. Significance refers to the results of post-hoc comparisons. ** *p* < 0.01. *n* = 6–8/group. For further details, see text.

Furthermore, while this drug failed to alter startle amplitude (Figure 4A), it restored PPI in D1CT-7 mice (Figure 4B). We then compared the effects of the most effective dose of DK-I-56-1 (10 mg/kg) on tic-like responses and PPI in comparison with those of the prototypical D_1_ and D_2_ dopamine receptor antagonists SCH 23390 (0.5 mg/kg, IP) and HAL (0.3 mg/kg, IP).

**Figure 5:**
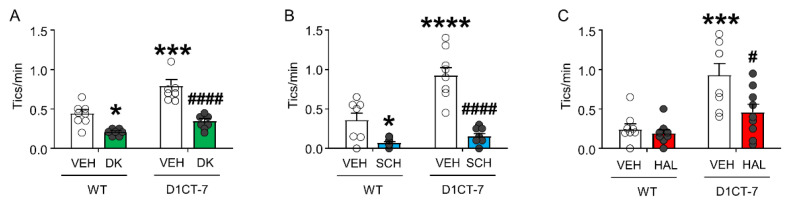
Effects of DK-I-56-1 (DK, 10 mg/kg, IP) on tic-like responses in D1CT-7 and wild-type (WT) mice exposed to spatial confinement, as compared with dopaminergic antagonists. (**A**) DK significantly reduced tic-like behaviors in both genotypes (Genotype × treatment interaction: F(1,25) = 4.46; *p* = 0.04). (**B**) The D_1_ receptor antagonist SCH 23390 (SCH, 0.5 mg/kg, IP) also reduced tic-like behaviors in all genotypes (Genotype × treatment interaction: F(1,32) = 11.54; *p* = 0.002). (**C**) The D_2_ receptor antagonist haloperidol had similar effects (HAL, 0.5 mg/kg, IP) (Genotype × treatment interaction: F(1,30) = 4.61, *p* = 0.04). All analyses were performed by two-way ANOVAs. * *p* < 0.05; *** *p* < 0.001; **** *p* < 0.0001 vs. WT mice treated with vehicle (VEH); ^#^ *p* < 0.05; ^####^ *p* < 0.0001 vs. D1CT-7 mice treated with VEH. *n* = 7–9/experimental group. For further details, see text.

As expected, DK-I-56-1 significantly reduced tic-like behaviors in both WT and D1CT-7 mice (Figure 5A). Tic-like responses were also reduced by the D_1_ receptor antagonist SCH 23390 (Figure 5B) and the D_2_ receptor antagonist HAL (Figure 5C).

**Figure 6:**
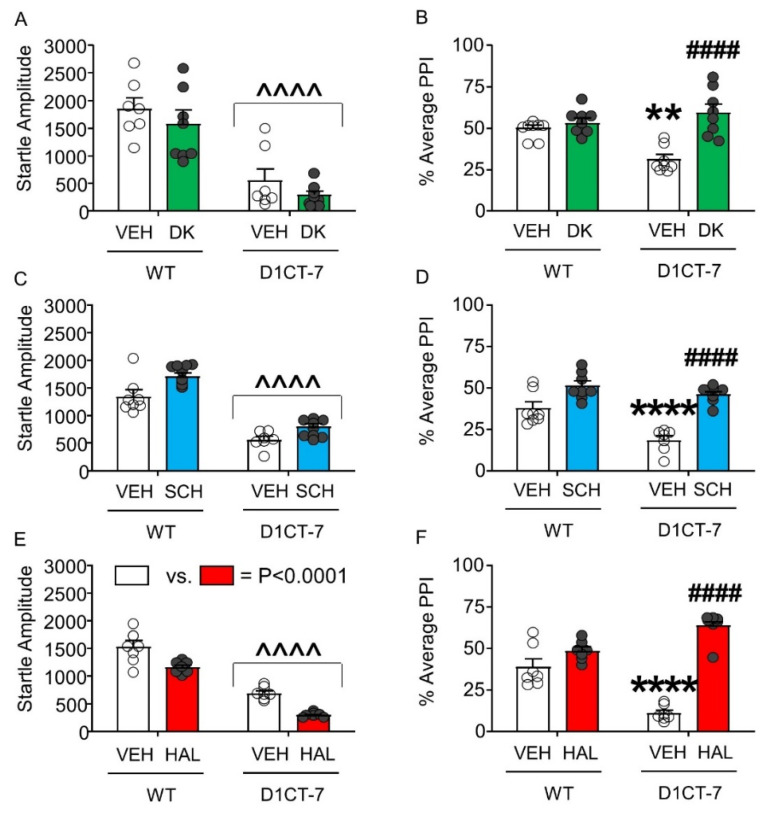
Effects of DK-I-56-1 (DK, 10 mg/kg, IP) and dopaminergic antagonists on tic-like responses in D1CT-7 and wild-type (WT) mice exposed to spatial confinement. (**A**) D1CT-7 mice had lower startle amplitude than WT littermates (Main effect of genotype: F(1,25) = 49.04, *p* < 0.0001), but this difference was not affected by DK. (**B**) DK reversed the PPI deficits observed in D1CT-7 mice, but not WT mice (Genotype × treatment interaction: F(1,25) = 12.37; *p* = 0.002). (**C**) The D1 receptor antagonist SCH 23390 (0.5 mg/kg, IP) did not affect startle amplitude. (**D**) SCH 23390 reversed the PPI deficits in D1CT-7 mice (Genotype × treatment interaction: F(1,24) = 5.36; *p* = 0.03). (**E**) The D2 receptor antagonist haloperidol (HAL, 0.5 mg/kg, IP) reduced startle reflex in both WT and D1CT-7 mice (Main effect of treatment: F(1,24) = 36.64, *p* < 0.0001). (**F**) HAL reversed the PPI deficits observed in D1CT-7 mice (Genotype × treatment interaction: F(1,24) = 44.99; *p* < 0.0001). ^^^^ *p* < 0.0001; main effect of genotype (D1CT-7 vs. WT); ** *p* < 0.01; **** *p* < 0.0001 vs. WT mice treated with vehicle (VEH); ^####^ *p* < 0.0001 vs. D1CT-7 group vehicle-treated. *n* = 7–8/group. For further details, see text.

Next, we analyzed the effect of DK-I-56-1, SCH 23390, and HAL on the startle response of D1CT-7 and WT mice. DK-I-56-1 did not alter the startle amplitude of either genotype, even though a significant reduction in startle reflex was found in D1CT-7 mice compared with WT littermates (Figure 6A). However, this drug reversed the PPI deficits observed in D1CT-7 mice without affecting this index in WT littermates (Figure 6B). Similar to DK-I-56-1, SCH 23390 did not affect startle amplitude (Figure 6C) but reversed the PPI deficits observed in D1CT-7 mice (Figure 6D). Finally, HAL was found to reduce startle amplitude in both genotypes, but this effect was not genotype-specific (Figure 6E). As expected, HAL reversed the PPI deficits observed in D1CT-7 mice (Figure 6F). 

**Figure 7:**
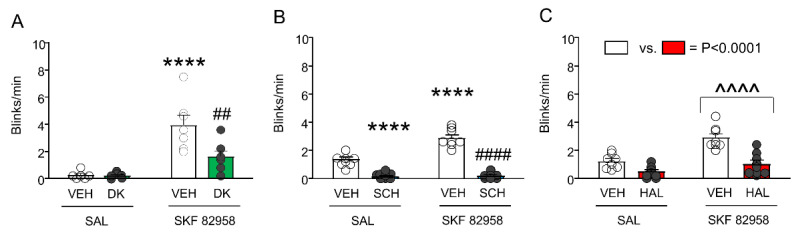
Effects of DK-I-56-1 (DK, 10 mg/kg, IP) and dopaminergic antagonists on the exacerbation of spontaneous eye blinking in C57BL/6 mice treated with SKF 82958 (0.5 mg/kg, IP). (**A**) Two-way ANOVA showed that SKF 82958 significantly increased eye blinking (Main effect: F(1,22) = 31.45, *p* = 0.00001), while DK reduced it (Main effect: F(1,22) = 6.75, *p* = 0.02). A significant interaction between the two treatments was found (F(1,22) = 6.37, *p* = 0.02), revealing that DK significantly reduced the blinks in mice treated with SKF 82958. (**B**) The D_1_ antagonist SCH 23390 (0.5 mg/kg, IP) also countered the elevation in eyeblinks caused by SKF 82958 (Main effect of SKF 82958: F(1,30) = 28.68, *p* < 0.00001; Main effect of SCH 23390: F(1,30) = 181.6, *p* < 0.00001; Interaction: F(1,30) = 25.10, *p* = 0.00002). (**C**) The D_2_ antagonist haloperidol (HAL, 0.5 mg/kg, IP) reduced eyeblinks, but this effect did not interact with the elevation of eyeblinks induced by SKF 82958. (Main effect of SKF 82958: F(1,28) = 22.98, *p* = 0.00005; Main effect of HAL: F(1,28) = 25.59, *p* = 0.00002; Interaction: F(1,28) = 2.79, NS). ^^^^ *p* < 0.0001 main effect of SKF 82958 vs. saline (SAL); **** *p* < 0.0001 vs. mice treated with VEH and SAL; ^##^ *p* = 0.001 and ^####^ *p* < 0.0001 vs. mice treated with VEH. *n* = 6–8/group. For further details, see text.

DK-I-56-1 counters the elevation of eyeblinks and PPI deficits induced by SKF 82958. We then tested the effects of DK-I-56-1 (10 mg/kg, IP), SCH, and HAL on the enhancement of eye blinking induced by SKF 82958 (0.5 mg/kg, IP) (Figure 7). As expected, SKF 82958 dramatically increased spontaneous eye blinking; furthermore, DK-I-56-1 reduced the number of eyeblinks. A significant interaction between the two treatments revealed that DK-I-56-1 significantly reduced the eyeblinks in SKF 82958-treated mice (Figure 7A). The analysis of SCH 23390 confirmed that this drug fully countered the elevation in eyeblinks caused by SKF 82958 (Figure 7B). Conversely, HAL reduced eyeblinks, but this effect did not interact with the elevation of eyeblinks induced by SKF 82958 (Figure 7C). 

**Figure 8:**
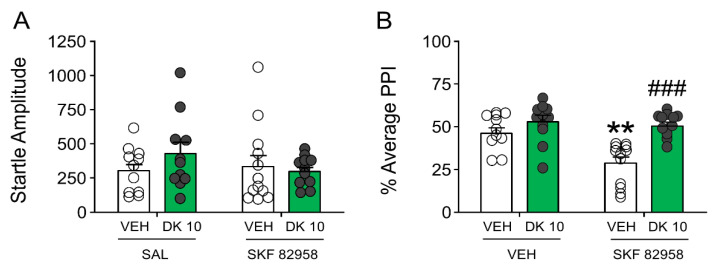
Effects of DK-I-56-1 (DK, 10 mg/kg, IP) on (**A**) startle response and (**B**) prepulse inhibition (PPI) in C57/BL6 mice treated with SKF 82958 (0.5 mg/kg, IP). Two-way ANOVA detected that SKF 82958 dramatically reduced PPI (Main effect: F(1,42) = 9.73, *p* = 0.003), and DK countered the PPI deficits caused by the D_1_ receptor agonist (Interaction: F(1,42) = 5.43, *p* = 0.02). Significance levels refer to the results of post-hoc comparisons. ** *p* < 0.01 vs. mice treated with the vehicle of DK (VEH) and saline (SAL, controlling for SKF 82958); ^###^ *p* = 0.0001 vs. mice treated with VEH and SKF 82958. *n*= 11–12/group. For further details, see text.

Building on these results, we then assessed whether DK-I-56-1 could reduce PPI deficits induced by SKF 82958 (0.5 mg/kg, IP) (Figure 8). The analysis of startle amplitude revealed that neither DK-I-56-1 nor SKF 82958 significantly modified this response (Figure 8A). Conversely, SKF 82958 dramatically reduced PPI; furthermore, DK-I-56-1 countered the PPI deficits caused by the D_1_ receptor agonist (Figure 8B). 

**Figure 9:**
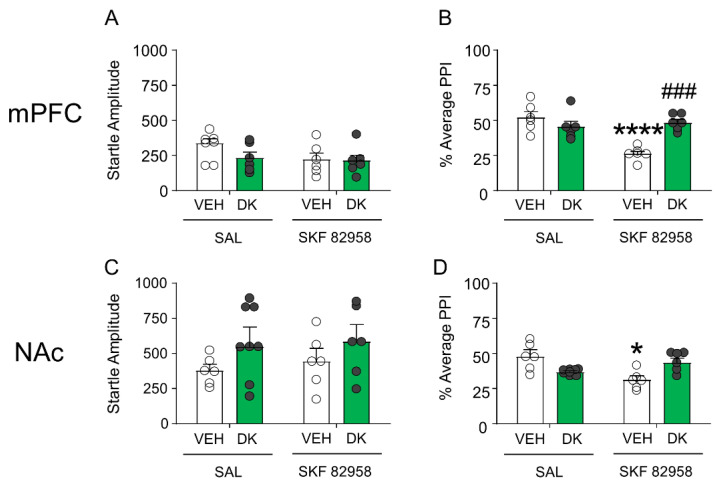
DK-I-56-1 (DK, 10 nmol) infusion in the medial prefrontal cortex (mPFC) (**A**) failed to affect startle reflex in mice, but (**B**) significantly countered the PPI deficits produced by SKF 82958 (F(1,20) = 19.34; *p* = 0.0003). Conversely, infusions of this drug in the nucleus accumbens (NAc) (**C**) did not affect startle amplitude, and (**D**) failed to reverse the PPI deficits induced by SKF 82958. All analyses were performed by 2-way ANOVAs. Significance levels refer to the results of post-hoc comparisons. * *p* < 0.05; **** *p* < 0.0001 vs. mice treated with the vehicle of DK (VEH) and saline (SAL, controlling for SKF 82958); ^###^ *p* = 0.0001 vs. mice treated with VEH and SKF 82958. *n* = 6–8/group. For further details, see text.

Given the postsynaptic location of D_1_ receptors, we inferred that the mechanisms of DK-I-56-1 might operate downstream from these receptors. Thus, we tested the brain-regional effects of this drug in two key regions implicated in the dopaminergic regulation of PPI, namely the medial prefrontal cortex and nucleus accumbens. As shown in Figure 9A,B, DK-I-56-1 (10 nmol) infusion in the medial prefrontal cortex failed to affect startle reflex in mice, but significantly countered the PPI deficits produced by SKF 82958 (F(1,20) = 19.34; *p* < 0.001; *p* < 0.01 for comparison of vehicle + SKF 82958 vs. DK-I-56-1 + SKF 82958). Conversely, no ameliorative effect was found when DK-I-56-1 was injected in the nucleus accumbens (Figure 9C,D).

DK-I-56-1 fails to induce catalepsy in the bar test. Finally, we tested the effects of DK-I-56-1 in the bar test. Unlike HAL, DK-I-56-1 failed to produce a significant increase in catalepsy (Table 1).

## 4. Discussion

The main results of this study indicate that DK-I-56-1, the selective PAM of α6 GABA_A_Rs, reduced tic-related responses in two distinct mouse models of TS, representing complementary aspects of tic phenomenology. While these effects were accompanied by ameliorative effects on the PPI deficits, they were not generalized to other behavior, underscoring the specificity of the effects of DK-I-56-1 to TS-pertinent behavioral manifestations. While the effects of DK-I-56-1 were similar to those of D_1_ and D_2_ receptor antagonists, the α6 GABA_A_R PAM did not elicit any extrapyramidal effects, as measured by catalepsy in the bar test. Taken together, these results strongly suggest that DK-I-56-1 may exert potent therapeutic properties in TS and other tic disorders with a good tolerability profile.

Several preliminary results point to a potential involvement of GABA_A_R α6 subunits in TS: for example, the gene *GABRA6* has been shown to affect TS risk [38]. Furthermore, the extract of the plant *Clerodendrum inerme* was found to reduce tic severity in a treatment-refractory case of tic disorder [39]. The main psychoactive ingredient of *C. inerme*, hispidulin, is also a PAM for α6 GABA_A_Rs and blocks the hyperlocomotion and PPI deficits induced by the potent dopamine releaser methamphetamine [15,40]. While PPI deficits induced by dopaminergic agents are used to detect potential therapeutic effects in TS [19], the lack of specificity of these phenotypes limits the predictive potential of these models.

The D1CT-7 mouse model of TS [21,22] was generated via a neuropotentiating transgene expressed in a subset of neurons harboring D_1_ receptors in the somatosensory cortex, piriform cortex, and intercalated nucleus of the amygdala [21]. The expression of this transgene results in several phenotypes strikingly reminiscent of TS, such as tic-like myoclonic axial jerks, which are attenuated by antipsychotics and other treatments approved for TS [22,23]. Most importantly, D1CT-7 mice also exhibit other phenotypic characteristics reminiscent of TS, including PPI deficits and a dramatic exacerbation of tic-like behaviors after exposure to spatial confinement, a mild acute stressor [23]. Our data indicate that α6 GABAARs control tic-like behaviors and PPI deficits in D1CT-7 mice. Although the mechanisms underlying these effects remain unknown, the enhancement of the inhibitory action in one of the neuropotentiated cortical areas in these models may be responsible for the observed responses. Of note, premonitory urges are associated with a reduction in gray matter in the somatosensory cortex and in the insula [41], possibly reflecting preliminary reports of reductions in interneurons [42] and alterations in the GABAergic tone [43]. Future studies are needed to verify whether potential GABAergic dysfunctions in these regions may be directly implicated in the genesis of premonitory urges and whether activation of α6 GABA_A_Rs may reduce these manifestations. 

Spontaneous eye blinking has long been considered one of the closest phenomenological proxies to tics. Indeed, both manifestations are typically sudden, semi-voluntary, and preceded by premonitory urges with similar neural substrates. Some studies have shown that TS patients have a higher frequency of eyeblinks in TS and report higher urge severity and discomfort in relation to eyeblink suppression. Previous studies have shown that spontaneous eyeblink is increased by activation of D_1_ but not D_2_ receptors [25]. Our data show that these responses are reduced by D_1_ and D_2_ dopamine receptor antagonists. These results underscore the high predictive validity of this model of TS.

It should be noted, however, that the effects of DK-I-56-1 were also observed in animals treated with saline solution, indicating that α6 GABA_A_Rs may control both spontaneous eye blinking activity as well as its exacerbation by D_1_ receptor activation. One of the most surprising findings of our study was that DK-I-56-1 reduced the PPI disruption mediated by the D_1_ receptor agonist SFK-82958, and local injections of this compound mimicked this effect in the medial prefrontal cortex. Although most α6 GABA_A_Rs are located on granule cells in the cerebellum [44,45,46], these receptors have also been documented in other brain regions of rodents, including the prefrontal cortex [47]. The enhancement of GABAergic tone in this region may be critical in improving information processing and sensorimotor gating by reinforcing lateral inhibition mechanisms. Future studies are warranted to substantiate further the mechanisms underlying our findings.

Several limitations of the present study should be acknowledged. First, our analyses were limited to two models of TS relying on a high face and predictive validity; however, our studies did not fully substantiate the mechanisms underlying the specific role of α6 GABA_A_Rs in TS pathophysiology and in the modulation of D_1_ receptor-mediated responses. Future studies will be needed on alternative models that address the relevance of these receptors with well-characterized elements of TS pathophysiology, such as the lack of striatal interneurons or the mutation of specific genes implicated in this disorder. Second, although our analyses reveal a potential involvement of the prefrontal cortex in the mechanisms of action of DK-I-56-1, our data cannot rule out the potential involvement of the cerebellum in these effects. Recent findings suggest a primary involvement of this region in the pathophysiology of TS [48,49,50]. In particular, recent studies point to abnormal discharges of cerebellar neurons in temporal proximity to tic execution [50]. From this perspective, it is also important to note that the cerebellum is connected to the basal ganglia via bidirectional connections [51,52]; moreover, tics are associated with aberrant activity in basal ganglia as well as enhanced cerebellar activity [50]. These premises support the possibility that activation of cerebellar α6 GABA_A_Rs may limit the expression of tic-like behaviors initiated in the striatum.

These limitations notwithstanding, our data strongly support the possibility that DK-I-56-1 may exert therapeutic properties in TS with limited liability for extrapyramidal symptoms. Given the current limitations of the available pharmacological armamentarium for TS management, our data encourage the further development of this drug and other α6 GABA_A_R PAMs as potential novel therapies for this disabling disorder.

## Figures and Tables

**Table 1:** Effects of DK-I-56-1, haloperidol on catalepsy in the bar test. *** *p* < 0.001 in comparison with DK-I-56-1.

Drugs	Catalepsy (s)	*n*
DK-I-56-1 (10 mg/kg, IP)	0.2 ± 0.04	8
Vehicle of DK-I-56-1	0.13 ± 0.05	6
Haloperidol (0.3 mg/kg, IP)	11.01 ± 1.2 ***^1^	8
Vehicle of haloperidol	0.3 ± 0.06	8

^1^*p* < 0.001 in comparison with vehicle.

## Data Availability

The data presented in this study are available on request from the corresponding author.
